# A Feasibility Study of an Intravascular Imaging Antenna to Image Atherosclerotic Plaques in Swine Using 3.0 T MRI

**DOI:** 10.1371/journal.pone.0108301

**Published:** 2014-09-26

**Authors:** Chen Zhang, Lei Zhao, Xiaohai Ma, Zhaoqi Zhang, Zhanming Fan

**Affiliations:** Department of Radiology, Beijing Anzhen Hospital, Capital Medical University, Beijing, China; University of Groningen, University Medical Center Groningen, Netherlands

## Abstract

**Purpose:**

To investigate the feasibility of an intravascular imaging antenna to image abdominal aorta atherosclerotic plaque in swine using 3.0T magnetic resonance imaging (MRI).

**Methods:**

Atherosclerotic model was established in 6 swine. After 8 months, swine underwent an MR examination, which was performed using an intravascular imaging guide-wire, and images of the common iliac artery and the abdominal aorta were acquired. Intravascular ultrasound (IVUS) was performed in the right femoral artery; images at the same position as for the MR examination were obtained. The luminal border and external elastic membrane of the targeted arteries were individually drawn in the MR and IVUS images. After co-registering these images, the vessel, lumen, and vessel wall areas and the plaque burden in the same lesions imaged using different modalities were calculated and compared. The diagnostic accuracy of intravascular MR examination in delineating the vessel wall and detecting plaques were analyzed and compared using IVUS.

**Results:**

Compared with IVUS, good agreement was found between MRI and IVUS for delineating vessel, lumen, and vessel wall areas and plaque burden (r value: 0.98, 0.95, 0.96 and 0.91, respectively; P<0.001).

**Conclusion:**

Compared with IVUS, using an intravascular imaging guide-wire to image deep seated arteries allowed determination of the vessel, lumen and vessel wall areas and plaque size and burden. This may provide an alternative method for detecting atherosclerotic plaques in the future.

## Introduction

Atherosclerotic cardiovascular disease remains the leading cause of death in developed countries [1]. The composition of atherosclerotic plaques plays a critical role in cardiovascular disease, prognosis, and thus, assessment of atherosclerosis is crucial [2], [3]. Along with the development of invasive and non-invasive imaging techniques, intravascular ultrasound (IVUS) is the most useful imaging modality to detect plaque [4]. However, IVUS is often limited by the sound-reflecting calcification associated with plaque [5], and outer vessel boundaries are lost because of acoustic shadowing. Even in the absence of significant calcification, IVUS may not accurately identify the outer arterial boundary, because it tends to blend gradually into the surrounding tissue without a distinct border [3].

Driven by the characteristics of high tissue resolution, magnetic resonance imaging (MRI) can overcome this problem and has shown great promise in assessing vessel walls; MRI has the potential to characterize both arterial atherosclerosis and aortic stenosis disease [6]. However, surface coil-mediated MR imaging is only suitable for imaging of superficially-located arteries, such as the carotid artery. Because the signal-to-noise (SNR) decreases as the distance between the target and the surface coil increase [7]. The advantage of vessel wall MR imaging is limited in deep-seated arteries, such as the abdominal aorta and iliac artery, thus, surface coil-mediated MR imaging is seldom used to evaluate plaques.

To solve this problem and obtain distinct features of deep vessel walls, different intravascular MRI (IVMRI) receiver coils have been developed [8]. IVMRI receiver coils provide better quality images and permit delineation of the fine structure of deep-seated arteries because of the proximity of the MR detector coil to the arterial wall.

The purpose of this study was to evaluate the deep vessel walls and plaques in an atherosclerotic model in swine by using of a modified high-resolution intravascular imaging catheter and compare its performance using IVUS.

## Methods

### Animal Model Preparation

This study was carried out in strict accordance with the recommendations in the Guide for the Care and Use of Laboratory Animals of the National Institutes of Health. The protocol was approved by the Committee on the Ethics of Animal Experiments of Capital Medical University, Beijing, China.

**Figure 1:**
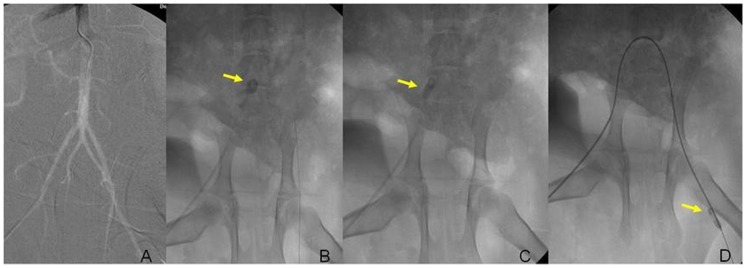
Digital subtraction angiography (DSA). It showed the abdominal aorta and bilateral iliac arteries of a miniature swine (A); the yellow arrow showed the balloon filling of water (B); ipsilateral iliac artery was injured using the balloon (arrow) (C); the contralateral iliac artery was injured (arrow) (D).

All surgery was performed under sodium ketamine anesthesia, and all animals were treated humanely. After intramuscular injection of sodium ketamine (2 mg/kg), a continuous intravenous infusion (1 mg/kg/h) was administered. At the end of the experiment, all animals were euthanized. Animals were euthanized by injection of potassium chloride. Six Guizhou miniature swine (15–20 kg) were included in the experimental protocol. Aortic wall injuries were induced using an intravascular balloon after the swine were fed an atherogenic diet for 2 weeks. After two weeks, animals were anesthetized with ketamine (1 mg/kg), and then balloon-induced aortic wall injury was performed using a 4–5F Fogarty catheter (Baxter Healthcare Corp, USA), which was introduced through the right femoral artery. The proposed Intravascular Loopless Monopole Antenna (ILMA) was first advanced to the abdominal aorta, the balloon was inflated with 5 ml saline, and the ILMA was then retracted down to the iliofemoral artery (Figure 1). This procedure was repeated 3 times in each animal. The ILMA was then removed, and the incision was sutured closed. After endothelial denudation, the animals were fed a high-cholesterol diet alternating with a regular diet.

### *In vivo* experiment

The ILMA consisted of an unshielded, flexible, low friction guide-wire and a connected matching/tuning circuit. The ILMA, which was used as a receive-only probe, was 0.48 mm in diameter and 58.7 mm in length. The guide-wire was made of nonmagnetic, anticorrosive and biocompatible metal [9].

For placement of the ILMA, the right femoral vein was surgically prepared and sectioned. The ILMA was placed into one side of femoral vein, iliac vein and inferior vena cava to avoid the artifacts caused by ILMA, which may reduce the image quality in the targeted artery. All MRI was performed on a 3.0-T system (Signa HDx, GE Medical Systems, USA). Animals were placed in a supine position on the MR table. Commercially available phased array coils were placed at the front and back of the animal’s pelvis area and MR examination was first performed using a surface coil to obtain the gross images of the abdominal and bilateral iliac arteries. For ILMA, the balloon-mounted intravascular coil was used for signal reception. The same sequences were used for the ILMA and the surface coil. The scan range covered the abdominal aorta from the origin of the right renal artery level to 5 cm after the bifurcation of the iliac arteries. The examination consisted of 2-dimensional double inversion recovery fast spin echo T1-weighted axial images (DIR T1WI), fat-suppressed fast spin echo T2-weighted (FSE T2W) and proton density weighted imaging (PDWI). Subsequently, 0.1 mmol/kg Gd-DTPA (Magnevist, Schering, Germany) was injected through an ear vein. The DIR T1WI sequence was repeated immediately after injection of contrast agent. The MRI acquisition parameters are listed in Table 1.

**Table 1:** MR Scan Parameters.

	T2WI	DIR-T1W	PDWI
TR (ms)	3000	520	3000
TE (ms)	65.2	9	17.2
FOV (cm)	30×24	30×24	30×24
Slice (mm)	5	8	8
matrix	192×192	224×224	192×192
Nex	8	4	6
slice pacing	0	0	0
Bandwidth(kHz)	31.2	31.2	31.2

TR, repetition time; TE, echo time; FOV, Field of View.

The MR images were analyzed with using commercially available software (ADW4.2, Report Card 2.0, GE Medical, USA). The average aortic wall thickness was measured on the pre-enhanced DIR T1W images. The vessel wall borders were magnified and manually identified by two readers. Total vessel area (TVA) was defined as the area circumscribed by the adventitia vessel wall border; lumen area (LA) was defined as intraluminal area; vessel wall area (VWA) was defined as TVA minus LA. Plaque burden was defined as (vessel area – lumen area)/vessel area.

### IVUS examination

After puncturing the right iliac artery, we measured the distance of the ultrasound catheter by body surface to match the MRI slices with IVUS images. Scans were recorded using an automatic retracement system, and the following definitions were used: slices that showed the origin of right renal artery were level 0; slices that were directed to the caudal side were defined as positive levels, and slices that were directed to the cranial side were defined as negative levels. The vessel wall borders were manually identified by two readers. Each reader determined whether the IVUS still frame was adequate for analysis (a visible external elastic membrane for at least 270°), and whether ILMA imaging could be analyzed.

### Statistical analysis

Analyses were performed using the SPSS 13.0 software package (SPSS, Chicago, IL). Continuous values are expressed as the means ± SD. An independent sample t-test was used to compare continuous data for between-group differences, the comparison of TVA, LA, VWA and plaque burden between ILMA and IVUS examination were statistically evaluated using paired t-tests. A linear regression and Bland-Altman analysis were performed to determine the correlation and limits of agreement between the two different imaging methods for vessel delineation.

## Results

### Animal models

The denudation procedure in the righty iliac artery failed in one animal because of severe stenosis, which was demonstrated by pathological findings, so left iliac artery denudation was performed in this animal. After 8 months, atherosclerotic plaques had developed on the aortic wall. Lumen stenosis and arterial wall irregularities were found on gross anatomical examination.

### Comparison between ILMA and IVUS

There were 180 MR image slices from the 6 swine, Forty-one slices had poor image quality because of motion artifacts and ILMA-related artifacts.

**Figure 2:**
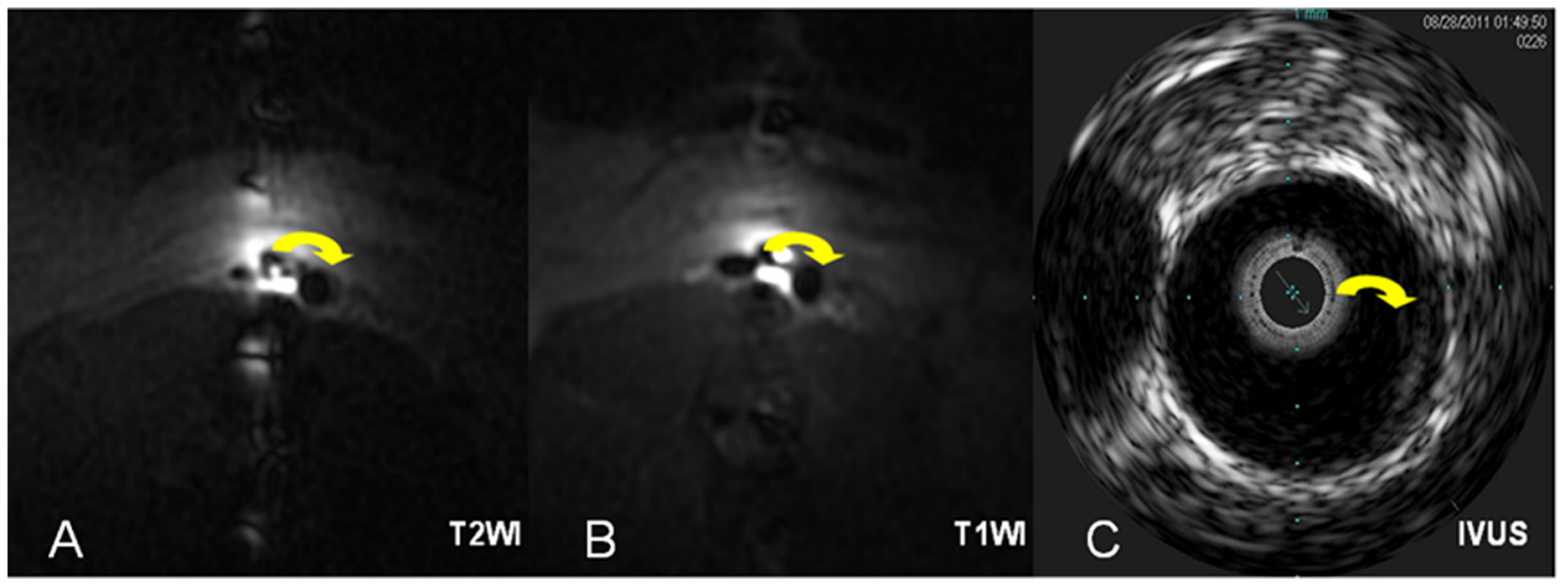
The matched image of ILMA and IUVS. Axial ILMA images of the abdominal artery using T2WI (A) and T1WI (B) and the corresponding IVUS iso-echo (C) demonstrate the accurate definition of plaque morphology (arrows reveal the thickened wall).

Thus 139 slices that contained no or mild artifacts were used for analysis, including 103 MR images slices with plaques and 36 slices without plaques (which also included slices of thickened vessel walls). No plaque rupture or thrombosis occurred during MR and IVUS examinations. (Figure 2).

**Figure 3:**
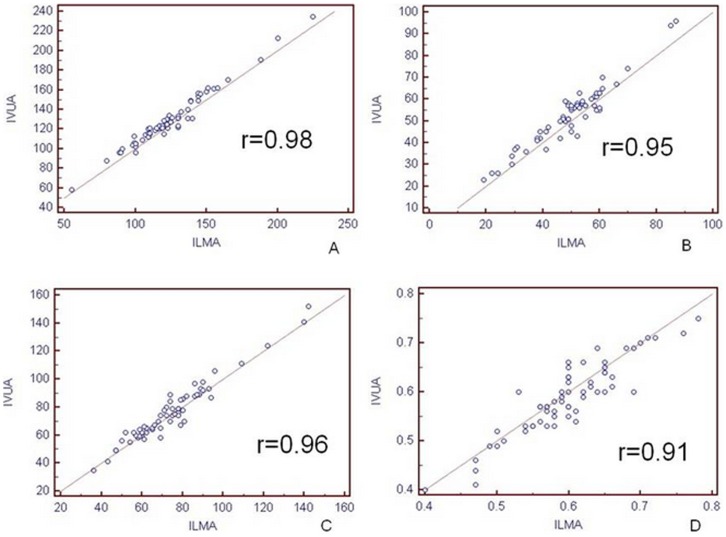
Correlations between ILMA and IVUS. The measurements of artery total vessel area (TVA, A), lumen area (LA, B), vessel wall area (VWA, C), and plaque burden (D) demonstrate that ILMA accurately determines plaque size and burden.

There was good correlation between ILMA and IVUS compared with vessel area, lumen area, vessel wall area and plaque burden (r values: 0.98, 0.95, 0.96, and 0.91, respectively; P<0.001; Figure 3).

**Figure 4:**
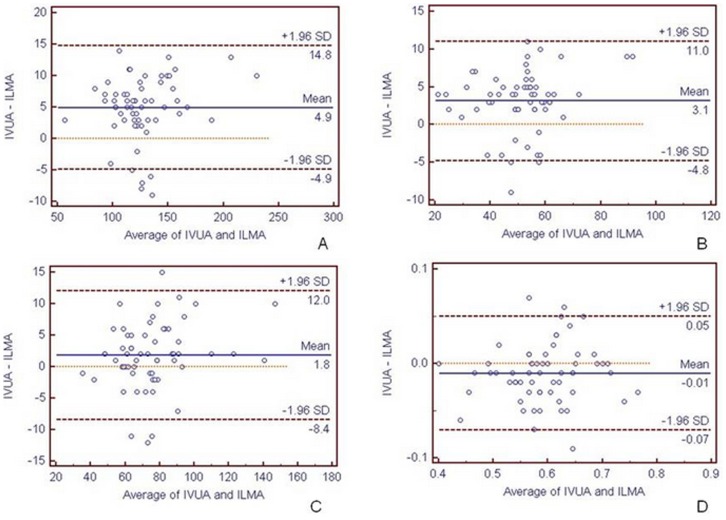
Consistency analysis diagram of ILMA and IVUS. Consistency of ILMA and IVUS measurements of artery total vessel area (TVA, A), lumen area (LA, B), vessel wall area (VWA, C), and plaque burden (D).

IVUS and ILMA were compared using the Bland-Altman analysis (Figure 4). For an average TVA of 124.08 mm^2^, the mean difference was 4.93 mm^2^ (limits of agreement, 4.93±9.81 mm^2^). For an average LA of 49.72 mm^2^, the mean difference was 3.13 mm (limits of agreement, 3.13±7.89 mm^2^). For an average VWA of 74.37 mm^2^, the mean difference was 1.8 mm (limits of agreement, 1.8±10.1 mm^2^). For an average plaque burden of 0.60 mm^2^, the mean difference was −0.03 mm^2^ (limits of agreement, −0.03±0.05, mm^2^). Compared with IVUS, the vessel area, lumen area, and vessel wall area measured using ILMA were smaller, but the plaque burden was larger, and the differences were statistically significant (P<0.05; Table 2).

**Table 2:** Comparing measurement using IVUS versus ILMA.

	TVA(mm^2^)	LA(mm^2^)	VWA(mm^2^)
ILMA	124.08±27.62	49.72±12.76	74.37±20.00
IVUS	129.02±28.53	52.85±13.45	76.17±21.14
t	7.62	6.01	2.67
P	P<0.001	P<0.001	0.01

TVA, total vessel area; LA, lumen area; VWA, vessel wall area.

Data are presented as the mean ± SD.

## Discussion

In this research, we evaluated the utility of a high-resolution receive-only intravascular loopless MR detector as a possible guidewire for identifying imaging and measuring the lumen area, vessel wall area and plaque burden using a widely-available clinical 3T MR system. In addition, compared with IVUS, our results demonstrated that there was a good correlation between MR and IVUS in measuring the lumen area, vessel wall area and plaque burden. Another main finding included establishment of an advanced atherosclerosis plaque model in swine which is similar to human atherosclerosis.

There are several intravascular MR detector coils designs including some that incorporate a loop or a solenoid, and also a loopless detector coil [10], [11], [12]. These loop detector coils have many clinical applications. For example, loop detectors are used to examine the prostate.

The proposed ILMA used in our experiments consists of a non-shielded flexible loach guide-wire and a connected matching/tuning circuit. The ILMA permits imaging over longer segments. The guide-wire was made of nonmagnetic, anticorrosive and biocompatible metal. And other advantages included a longer longitudinal coverage and a relatively smaller artifact. This coil was 0.48 mm in diameter and 58.7 mm in length, and the ILMA longitudinal coverage allows multi-slices scanning in large animal models during the same examination. Some types of intravascular MR device can be built as thin as 0.014 inches [13], [14] in diameter, which can be used in the coronary artery, However, the performance of this thinner intravascular MR coil needs further validation.

Some research on the superficial arteries, such as the carotid artery, has used a superficial array coil that can acquire the pathological features of the plaque. A previous study [15] has demonstrated the feasibility of using intravascular MR to visualize the vessel wall of deep-seated arteries. In the present study, we modified the ILMA to minimize motion and susceptibility artifacts near to the target artery, and inserted the guide wire into the veins adjacent to the abdominal arteries.

MR offers intrinsically high tissue resolution that adds unique value and provides intravascular imaging without X-ray guidance or ionizing radiation, However, when at clinical field strengths of B0 = 1.5 Tesla (T), MR spatial resolution remains a challenge, and spatial resolution is low for tiny vessels, such as coronary arteries. However, the recent emergence of clinical 3T MR scanners affords new opportunities to solve these problems because of an almost quadratic gain in the signal-to-noise ratio (SNR) with B0, A study [16] demonstrated an approximately 4-fold higher SNR and an approximately 10-fold increase in the visual field-of-view at 3 T compared to the original 1.5T.

The results of our study revealed good agreement between intravascular MR and IVUS in vessel measurement. Although there was good correlation between IVUS and intravascular MR in vessel measurement in the present study, ILMA underestimated TVA, LA and VWA compared with IVUS, Potential explanations maybe the differences in spatial resolution between the two techniques (intravascular MR coil, 0.03 mm; IVUS, 0.01 mm); and the calcium component in plaques, because IVUS cannot accurately measure calcified components, Thus, there would be bias in measurement bias.

The advantage of MRI is to differentiate between tissue components using multiple sequences [3]. Different components in the plaques would present different signal intensity according to the specific sequence used. Because extensively calcified regions contain few water protons, calcified plaques appear as signal voids in MR images. However, calcification interferes with measurement of atheroma size using IVUS, because outer vessel boundaries are lost because of acoustic shadows [3]; calcification also contributes to such difficulties in interpreting intravascular MR.

## Limitation

Our study has some limitations. We did not analyze the plaque components and further research is needed to validate the ability of ILMA in assessing specific plaque types; Moreover, the image quality of intravascular MR images is relatively poor, and further modification of ILMA is needed before it can be used in the clinical practice; In addition, heating at the tip of the wire during radiofrequency transmission should be considered.

In conclusion, compared with IVUS, intravascular MR can accurately delineate plaque size in deep-seated arteries. As this technology continues to be improved, intravascular MR should evolve into an important investigative and clinical tool.
